# Supplementary material for: Many-body screening effects in liquid water
Source: Nat Commun. 2023 May 11;14:2705. doi: 10.1038/s41467-023-38420-w (PMC10175292; doi:10.1038/s41467-023-38420-w)
Supplement: Supplementary file 1 — Supplementary Information [file 41467_2023_38420_MOESM1_ESM.pdf]

Supplementary Information for

Many-body screening effects in liquid water

Igor Reshetnyak[1], Arnaud Lorin[1], Alfredo Pasquarello[1*]

[1] *Chaire de Simulation à l'Echelle Atomique (CSEA), Ecole Polytechnique Fédérale de Lausanne (EPFL), CH-1015 Lausanne, Switzerland*

These authors contributed equally: Igor Reshetnyak, Arnaud Lorin

[*]Corresponding author, email: alfredo.pasquarello@epfl.ch.

Supplementary Methods

Convergence study for the qs*GW* calculations. We have performed a convergence study of the qsGW calculation of the band gap with respect to the number of updated states, the total number of states, and the cutoff used in the correlation part of the selfenergy for one configuration of the liquid. The corrections are determined with respect to the result obtained for the production setting of the parameters (see Supplementary Figure 1). The production calculations are performed with 550 updated states, a total number of 2000 states, and a cutoff of 12 Ry in the correlation part of the selfenergy. To extrapolate the corrections, we use linear functions in the inverse number of total states and in the inverse number of updated states. For the energy cutoff in the correlation part of the selfenergy, we use an exponential function. We estimated respective corrections of 0.32, 0.10, and 0.01 eV. These corrections are included in the final results.

Convergence study for the time-dependent density functional theory calculations. Convergence studies for the imaginary part of the inverse dielectric function at $q = 0.32$ Å^{-1} using time-dependent density functional theory (TDDFT) are given in Supplementary Figure 2. Panel **a** shows the convergence with the total number of bands included in the calculation, panel **b** shows the convergence with the energy cutoff used for defining the expansion of the wave functions, and panel **c** shows the convergence with respect to the energy cutoff for the dielectric matrix. The figures show that the curves are converged for the parameters chosen in our calculations (total number of band of 1428, cutoff of 41 Ry for the wave functions, and cutoff of 6 Ry for the dielectric matrix).

Supplementary Discussion

Dielectric function obtained with time-dependent density functional theory. We calculate the imaginary part of the dielectric function ϵ_2 for the set of values for α and β adopted in the main text ($\alpha = -10$ a.u. and $\beta = -0.2$ a.u.). In Supplementary Figure 3, we compare the calculated ϵ_2 with available experimental curves. While these parameters have been set to reproduce the loss function at $q = 0.32$ Å^{-1}, we remark that they also provide a reasonable description of ϵ_2, viz. the location of the main peak at ≈ 13 eV and the shoulder at ≈ 20 eV fall close to the positions of the corresponding experimental features. However, the TDDFT spectrum is found not to reproduce the detailed shape of the experimental spectrum near the onset due to the lack of excitonic effects. Despite the absence of these features, the TDDFT calculation yields a loss function (cf. Figure 2 in the main text) in good agreement with experiment. Thus, the comparison in Supplementary Figure 3 gives us further confidence in the description provided by the f^*_{xc} kernel.

Next, in order to optimise α and β from the comparison with the experimental curves for ϵ_2, we study in Supplementary Figure 4 how variations of α and β affect the calculated ϵ_2. Upon variation of α, the height of the first peak in ϵ_2 varies strongly, but the height of the shoulder remains unaffected. Furthermore, we observe a global shift towards higher energies with increasing α. The variation of β mainly influences the height of the first peak and to a lesser extent the height of the shoulder, with a dependence on β that progressively vanishes at higher energies.

Taking advantage of these trends, it is possible to identify a set of values for α and β that futher improve the comparison of the calculated ϵ_2 with its experimental counterpart, as shown in Supplementary Figure 3 when setting $\alpha = -8$ a.u. and $\beta = -0.185$ a.u. Indeed, for this choice of α and β, the height of the first peak is found to be noticeably improved. As a final check, we use the values of α and β obtained from the optimisation of ϵ_2 ($\alpha = -8$ a.u. and $\beta = -0.185$ a.u.) for the re-evaluation of the loss function at $q = 0.32$ Å^{-1} and $q = 0.95$ Å^{-1} (cf. Supplementary Figure 5). In Supplementary Figure 5, we compare the calculated curves with both the result presented in the main text (obtained with $\alpha = -10$ a.u. and $\beta = -0.2$ a.u.) and with the experimental curve from Hayashi & Hiraoka [1], finding satisfactory agreement. These results indicate that the description of the

global dielectric function is overall well described with one specific choice of α and β and that it remains robust upon small variations of these parameters.

Time-dependent density functional theory vs. Bethe-Salpeter equation. In Supplementary Figure 7, we show on the same scale the absorption spectra obtained by solving the Bethe-Salpeter equation (BSE) on top of qs$G\tilde{W}$ and qsGW, in comparison with the TDDFT absorption spectrum obtained through the two-point susceptibility including the f_{xc}^{*} kernel. The experimental spectrum obtained by Heller et al. is also reproduced for convenience. From Supplementary Figure 7, it clearly appears that the TDDFT spectrum does not reproduce the detailed shape of the experimental spectrum near the onset, lacking the excitonic contributions. However, the TDDFT calculation is sufficiently accurate to provide a good description of the loss function Im[ϵ^{-1}] over an extended range of frequencies, as can be seen in Figure 2 of the main text.

Supplementary Figures

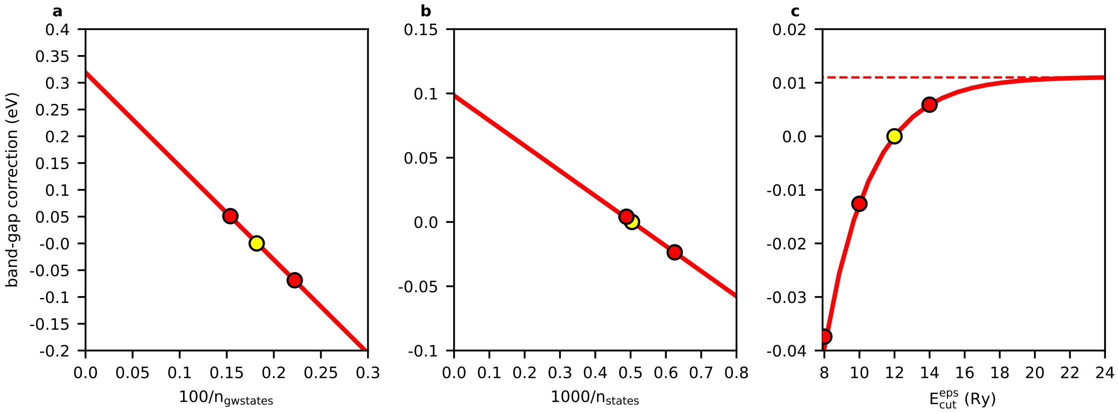

Supplementary Figure 1: Convergence study of the quasiparticle selfconsistent GW band gap. The band gap is studied as a function of **a** the number of updated states n_{gwstates}, **b** the total number of states n_{states}, and **c** the cutoff $E_{\text{cut}}^{\text{eps}}$ used in the correlation part of the selfenergy. The yellow disks indicate the production settings. The horizontal red line (dashed) in **c** corresponds to the converged value resulting from a fit to an exponential function.

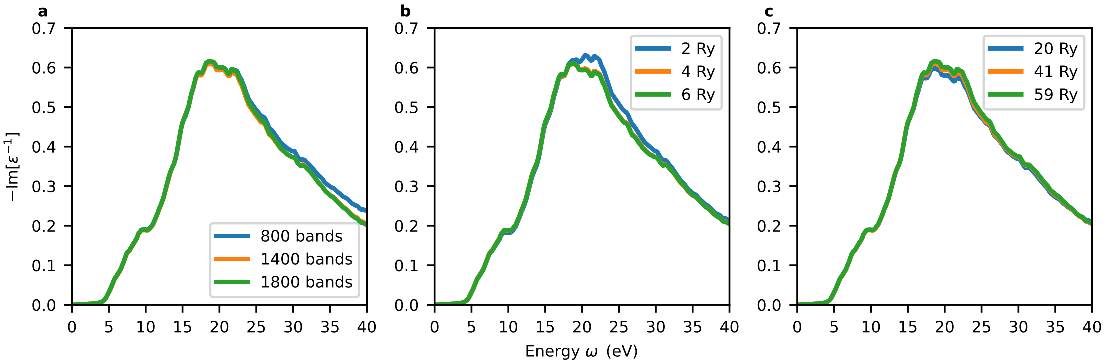

Supplementary Figure 2: Convergence of the loss function. The loss function $-\mathrm{Im}[\varepsilon^{-1}]$, where ε is the dielectric function, is given as a function of the energy ω, upon variation of **a** the total number of bands, **b** the energy cutoff defining the expansion of the wave functions, and **c** the energy cutoff of the dielectric matrix.

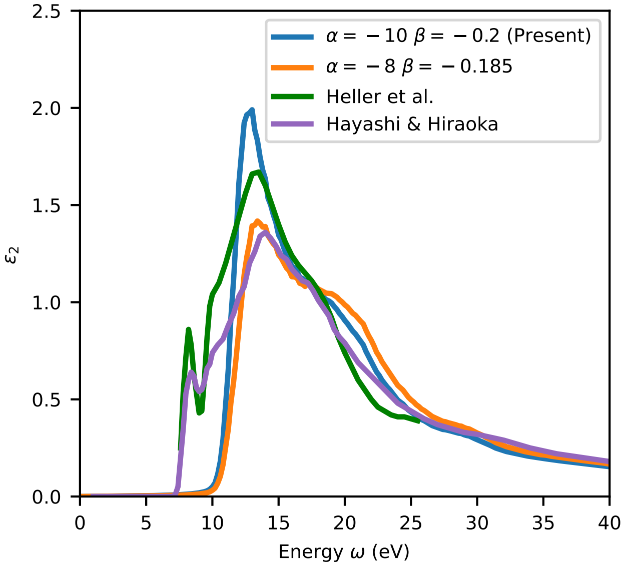

Supplementary Figure 3: Imaginary part of the dielectric function ϵ_2 for two sets of parameters. ϵ_2 is obtained by solving the time-dependent density functional theory (TDDFT) equation with the kernel f^*_{xc} for different values of α and β. The results are averaged over configurations of liquid water. Experimental curves from Heller et al. [2] (purple) and from Hayashi & Hiraoka [1] (green) are shown for comparison.

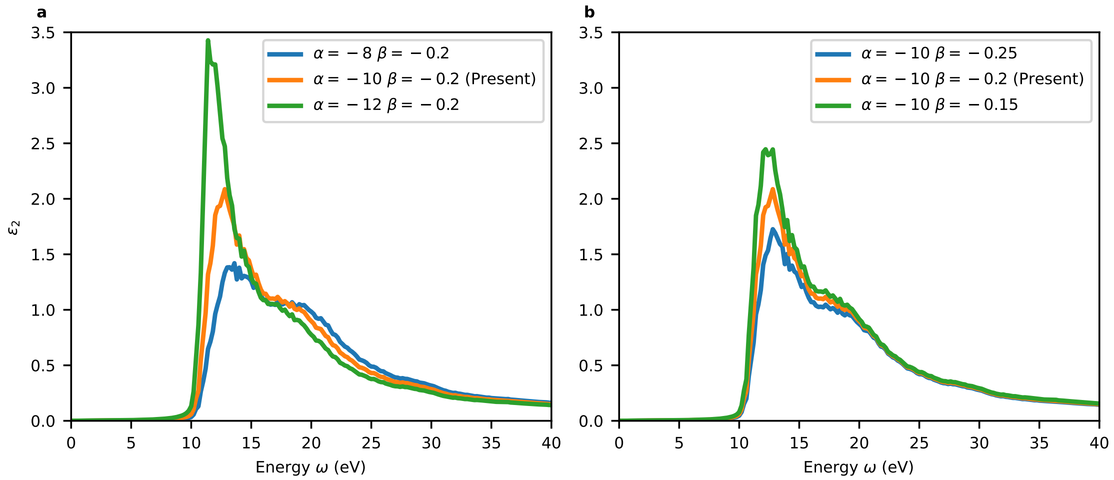

Supplementary Figure 4: Dependence of the imaginary part of the dielectric function ϵ_2 on α and β. ϵ_2 is shown upon variation of **a** α and **b** β in the kernel $f_{\rm xc}^*$, with respect to the parameters chosen in the main text ($\alpha = -10$ a.u. and $\beta = -0.2$ a.u.). The calculations are obtained for one representative configuration of liquid water.

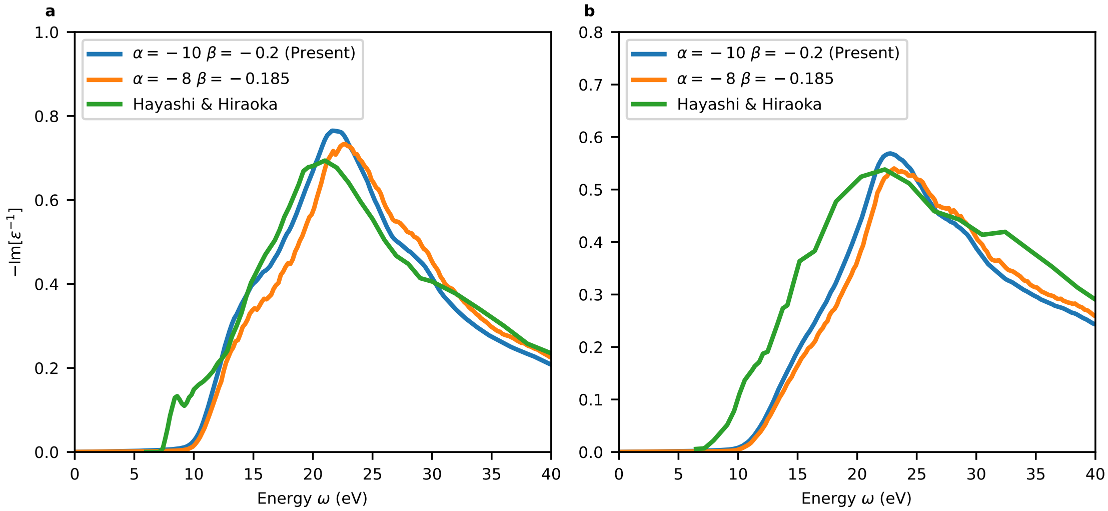

Supplementary Figure 5: Loss function at two different transferred momenta. The loss function $-\mathrm{Im}[\varepsilon^{-1}]$, where ε is the dielectric function, is shown at transferred momenta **a** $q = 0.32$ Å^{-1} and **b** $q = 0.95$ Å^{-1}, obtained by solving the time-dependent density functional theory (TDDFT) equation with the kernel f^*_{xc} for two sets of parameters α and β. The results are averaged over configurations of liquid water. Experimental curves from Hayashi & Hiraoka [1] (green) are shown for comparison.

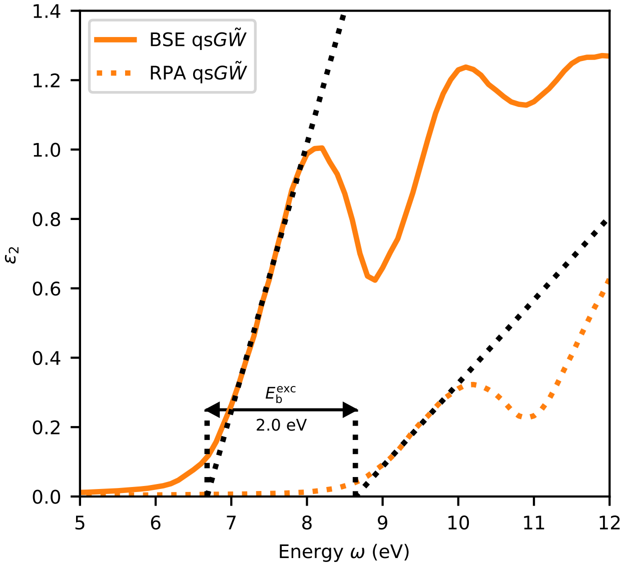

Supplementary Figure 6: Illustration of the exciton binding energy E_b^{exc}. The imaginary part of the dielectric functions ϵ_2, obtained with the Bethe-Salpeter equation (BSE) and with the random phase approximation (RPA), are shown as a function of the energy ω. The calculated spectra correspond to an average over the configurations of liquid water. E_b^{exc} is obtained from the separation between the onsets in the BSE and RPA spectra. The oblique dotted lines are tangential at the inflection points of the low-energy wings of the first peaks and define the onsets of the spectra. The present spectra are obtained with the qs$G\tilde{W}$ scheme, for which E_b^{exc} = 2.0 eV.

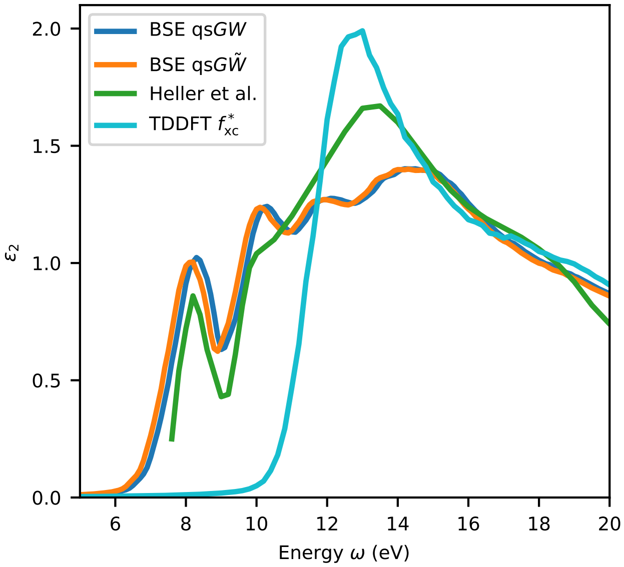

Supplementary Figure 7: Imaginary part of the dielectric function ε_2 as obtained with different theoretical schemes. The spectra obtained with the Bethe-Salpeter equation (BSE), based on quasi-particle selfconsistent GW without vertex corrections (qsGW) and with Bootstrap vertex corrections (qs$G\tilde{W}$), are compared with the spectrum obtained with time-dependent density functional theory (TDDFT) in which the f^*_{xc} kernel is used. The experimental spectrum obtained by Heller et al.[2] is also reproduced.

Supplementary References

1. Hayashi, H. & Hiraoka, N. Accurate measurements of dielectric and optical functions of liquid water and liquid benzene in the VUV region (1-100 eV) using small-angle inelastic X-ray scattering. *J. Phys. Chem. B* **119**, 5609–5623 (2015).

2. Heller, J. M., Hamm, R. N., Birkhoff, R. D. & Painter, L. R. Collective oscillation in liquid water. *J. Chem. Phys.* **60**, 3483–3486 (1974).
